# Nurses’ Experiences Using an Interactive System to Assess and Manage Treatment-Related Symptoms of Patients With Pancreatic Cancer: Interview Study

**DOI:** 10.2196/36654

**Published:** 2022-05-16

**Authors:** Maria Mangsbacka, Tina Gustavell

**Affiliations:** 1 School of Health and Welfare Dalarna University Falun Sweden; 2 Nursing Research Department of Surgical Sciences Uppsala University Uppsala Sweden; 3 Division of Nursing Department of Neurobiology, Care Sciences and Society Karolinska Institutet Stockholm Sweden; 4 Department of Upper Abdominal Diseases Cancer Theme Karolinska University Hospital (Huddinge) Stockholm Sweden

**Keywords:** app, health care professionals, mobile health, mHealth, nurses, pancreatic cancer, person-centered care, symptom-management, qualitative interview, nursing, interview

## Abstract

**Background:**

Treatment for pancreatic cancer entails symptom distress and a high burden of self-care. Patient-reported outcomes, collected with the support of mobile health (mHealth), have shown positive effects on symptom management, patient satisfaction, and quality of life for patients with cancer. For mHealth tools to become an integral part of clinical routine, experiences from health care professionals are needed.

**Objective:**

The aim of this paper is to describe nurses’ experiences of integrating an interactive system (Interaktor) for symptom assessment and management into daily practice, when caring for patients following pancreaticoduodenectomy and during chemotherapy treatment due to pancreatic cancer.

**Methods:**

Patients reported symptoms via the Interaktor app daily for 6 months. In the event of alarming symptoms, an alert was triggered to the patient’s nurse who then called the patient to offer advice and support. All nurses (n=8) who assessed patients were interviewed either individually or in a group. Transcribed interviews were analyzed using qualitative thematic analysis.

**Results:**

mHealth can facilitate person-centered care by offering nurses a way to gain knowledge about patients and to build relationships. Further, obstacles to implementation could be seen due to a lack of structural prerequisites and uncertainty about multiple ways to interact with patients.

**Conclusions:**

The Interaktor system can provide person-centered care. However, to implement mHealth tools as a clinical routine, focus needs to be placed on creating the necessary organizational conditions.

## Introduction

Pancreatic cancers have a high mortality rate and short median survival. The best hope of curing the cancer is to undergo surgical resection followed by adjuvant chemotherapy. The most common surgical procedure is pancreaticoduodenectomy where the head of the pancreas, duodenum, distal common bile duct, the gall bladder, and sometimes the gastric antrum and pylorus are removed. Unfortunately, the surgery often results in severe symptoms and a prolonged reduction in quality of life [1]. Common symptoms are related to eating, bowel function, and emotional well-being, along with fatigue and pain [2]. Great demands are placed on patients to manage their illness following surgery, and patients experience high levels of unmet physical and psychological supportive care needs [3]. Collecting electronic patient-reported outcomes (PRO) has an important role in supportive care to patients with pancreatic cancer [4]. Medical and public health practices supported by mobile devices have been defined by the World Health Organization as mobile health (mHealth) [5]. Several studies have shown positive effects on symptom management and quality of life when mHealth is used to collect and monitor PROs and to support self-management in cancer care [6,7].

The Interaktor system has been developed to meet the different needs patients may experience as they manage symptoms and concerns related to their illness. Interaktor includes a web interface and an app that is downloaded onto a smartphone or a tablet. The components are as follows: (1) patients’ assessment of the occurrence, frequency, and distress level of symptoms; (2) a web interface for the health care providers, for monitoring patients’ data in real time; (3) an alert function based on a risk assessment model, which sends alerts to the nurses via SMS text messaging; (4) access to evidence-based self-care advice related to symptoms and links to relevant websites; and (5) graphs of symptom report history. The web interface functions both as an aid in patient-clinician communication about symptoms and self-care and as a decision aid for health care professionals to manage symptoms. The reported data are stored on a designated secure server [8]. The Interaktor app has been evaluated for patients with breast-, pancreatic-, or prostate cancer with positive effects on symptom burden [9-11]. Patients with pancreatic cancer described that the app enabled them to be seen as a person by allowing their voices to be heard, to have access to an extended arm of health care, and to learn about their own health [12].

In Sweden, patients with a cancer diagnosis are offered a nurse navigator. The nurse navigator is a registered nurse (3 years of higher education with a bachelor’s degree) who functions as a support for the patients and relatives throughout the care chain. The role includes being accessible, informing about future steps in care and treatment, providing support in the event of normal crisis reactions, and mediating contacts with other professional groups [13].

The benefit to patients with cancer of using mHealth to interact with health care is clear. However, there is limited research regarding health care professionals’ perspective on how mHealth can enhance care and be implemented as clinical practice. To implement mHealth as clinical practice, there is a need to gain further knowledge about how the use of mHealth can be helpful for health care professionals in their work and about requirements for implementation. Therefore, the aim of this study was to describe nurses’ experiences of integrating an interactive system (Interaktor) for symptom assessment and management into daily practice when caring for patients following pancreaticoduodenectomy and during chemotherapy treatment due to pancreatic cancer.

## Methods

### Design

This study has a qualitative descriptive design.

### Setting, Procedure, and Participants

The study was performed at a high-volume center for pancreatic surgery, with over 100 pancreaticoduodenectomies performed annually. At the time of the study, standard care after discharge following pancreaticoduodenectomy and during adjuvant chemotherapy meant that patients were able to call the clinic’s advice line during office hours (daytime, Monday to Friday) to contact their nurse navigator when necessary. A few weeks after discharge, patients had one scheduled visit to the surgeon and sometimes the nurse navigator where symptoms could be discussed. Patients undergoing adjuvant chemotherapy were monitored weekly before the start of the intravenous treatment. Patients with severe symptoms were referred to an advanced home care team offering frequent visits by nurses at home.

In addition to standard care, patients using the Interaktor app reported symptoms to their nurse navigator, starting the first day after discharge and until 1 week after their final dose of intravenous chemotherapy. They were encouraged to report symptoms once a day, preferably in the morning. They were informed that alerts were only monitored during office hours, and if they needed help at other times, they were to telephone the national Healthcare Advice Line or visit the nearest Emergency Department. The nurse’s involvement was to monitor alerts and call patients following an alert to give support. The alerts came as an SMS text message to a study-specific mobile phone, one at each unit. For red alerts, a nurse should contact the patient within 1 hour. For yellow alerts, contact should be made within the same day.

Before the study started, the nurse navigators were invited to attend a 2-hour training session on the use of the Interaktor platform and their responsibilities throughout the study. Nurse navigators who could not attend the training session, or those employed after the session, received “in-house” training from nurses who had attended the training session. The training was held by one of the researchers, and throughout the study, the same researcher was available to answer questions that might arise. The total study period was 21 months. During that time, the nurse navigators at the surgical clinic monitored all patients who started to use the app (n=36), and they had 1-10 patients to monitor at the same time. Not all patients received adjuvant chemotherapy treatment. Those who did (n=21) were allocated to one of two oncological clinics. Those two clinics had, respectively, 1-8 patients and 1-5 patients to monitor at the same time.

### Sample

All nurse navigators (n=8, hereafter referred to as nurses) who had monitored symptom alerts of patients with pancreatic cancer using the Interaktor app were invited to participate in the study, and all consented. The nurses worked either at the surgical clinic where they monitored patients after discharge and until start of adjuvant chemotherapy, or at one of the oncological clinics where the patients received their chemotherapy for up to 6 months.

### Data Collection

Focus groups were chosen since this method takes advantage of group processes to explore and clarify participants’ attitudes, needs, and ideas of structural solutions [14]. The focus groups (n=3) were held at the workplace during working hours with nurses from the same unit, to take advantage of natural interactions. One nurse was interviewed over the phone since she was unable to participate in the focus group with her colleagues. To ensure trustworthiness, the interviews followed a semistructured interview guide (Textbox 1). The interviews were audio recorded and lasted for 18, 22, 23, and 25 minutes, respectively.

**Semistructured interview guide used for the focus groups (n=3) and the individual interview (n=1).**

**Main questions**

- What was it like to implement the app system?
- How did it affect your working situation?
- What was it like to monitor alerts?
- How well did the technology function?
- Can you describe in which way the patients using the app contacted you during the study and if there were any differences between them and other patients?
- What were your thoughts about the benefits for patients who used the app?

### Data Analysis

**Figure 1:**
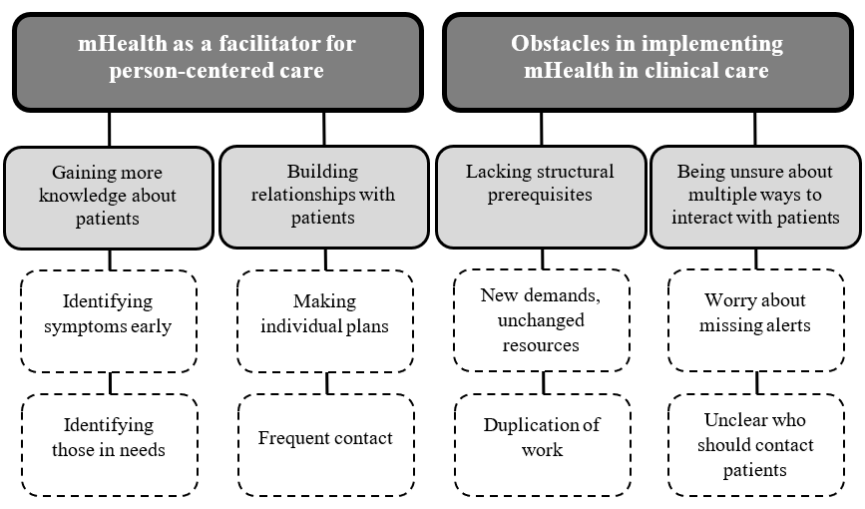
Examples of areas (white rectangle) connected to sub-themes (light grey rectangle) and overarching themes (dark grey rectangle) identified through the thematic analysis of interviews with nurses (n=8) responsible for monitoring and responding to alerts coming from patients using the Interaktor app following pancreaticoduodenectomy due to cancer.

The interviews were analyzed using thematic analysis [15]. To begin with, the first author transcribed all interviews verbatim, and both authors read through the transcripts several times to familiarize themselves with the data. Then, the text was systematically coded throughout the entire data set with an inductive approach. A code consisted of a few words or a sentence related to the aim of the study. After the initial coding, matching codes were put together into areas. The areas were then reviewed so that they covered all codes. After this step, the areas were analyzed into themes. The themes were reviewed, discussed, and revised several times by both authors. The final analysis resulted in 2 main themes with 2 subthemes each (Figure 1). Some quotes were chosen to exemplify the findings, which are presented in the Results section. To establish rigor of the analysis, the 15-point checklist of criteria for good thematic analysis by Braun and Clark [15] was followed.

### Ethical Considerations

Ethical approval was given by the Regional Ethical Review Board in Stockholm, Sweden (Registration number 2011/1780-13/2), and the research followed the Declaration of Helsinki. Before inclusion in the study, all participants were informed, both verbally and in writing, about the voluntary nature and confidentiality of participation and their right to withdraw at any time. They were also made aware that confidentiality would be preserved and that access to the audio recordings would only be given to researchers involved in the analysis. Further, they were informed that quotes from the interviews would be formulated to protect the identity of the participants.

## Results

The two main themes identified were as follows: “mHealth as a facilitator for person-centered care” and “Obstacles in implementing mHealth in clinical care.”

### mHealth as a Facilitator for Person-Centered Care

#### Gaining More Knowledge About the Patients

The nurses described that through the Interaktor system, they could capture patients who did not normally contact them. Even though they told their patients to call if distressing symptoms occurred, they were aware that some patients hesitated to call since they did not want to bother the nurse. In the nurses’ experiences, many patients normally waited a long time before contacting their health care provider. The alerts enabled the nurses to be notified right away if a patient had symptoms at home that needed attention. Thereby, even patients who hesitated to call were acknowledged and could receive rapid support, and attention could be focused on patients most in need.

Some might think to themselves, okay, I’ve got a fever but it’s probably nothing to worry about, even though we've told them to call if they have a fever. The system allows us to automatically call in the event of an alarm, I think that's the most important thing. Then, hopefully, you can spend time on the patients with problems, rather than calling the ones who are well and don't really need any help.

Nurse at Unit C

The nurses mentioned that the alerts and their different response times contributed to the rapid reaction. The early identification of symptoms and the possibility to view symptom graphs enabled the nurses to check on their patients at home. From the nurses’ perspective, the patients felt secure and relaxed knowing that someone was monitoring them and that someone other than themselves was responsible for them being contacted.

The patients could rely on us to contact them, meaning that they did not need to feel responsible for contacting us. They trusted that we would contact them when they reported more severe symptoms. I really think they appreciated that.

Nurse at Unit A

#### Building Relationships With Patients

The nurses stated that they had more regular contact with the patients who used the app and therefore spent more time with them. Thus, this experience showed the nurses that patients felt close to them even if the patients were at home.

The alert levels did not suit all patients, which resulted in alerts being triggered even if the patient was not in need of support. Patients sometimes felt guilty and apologized if the nurse called several times for the same alert. As a solution, the nurses made individual plans together with the patients about when to call and when not to call. In addition, the patients were encouraged to write messages in the free text section to specify if they needed contact or not. This enhanced the communication between the nurse and the patient.

And then there was a patient who we had a lot of contact with. She had numbness all the time and felt that many times I called unnecessarily. But after a few times I still wanted to call, but then she said, I will write a comment if I want you to call. That was --- [mentions patient by name], I think you knew her even better [addresses colleague].

Nurse at Unit C

The self-care advice enabled the nurses to give personalized advice to the patients. When talking to a patient, the advice could form the basis of the conversations. The nurses and the patients could then discuss different actions and what suited that patient, or the nurse could clarify the text that the patient had read. Further, by reading the self-care advice, patients could ease their symptom burden at home without having to seek medical care, and self-control of the disease could be achieved, which created security.

### Obstacles in Implementing mHealth in Clinical Care

#### Lacking Structural Prerequisites

The nurses felt that they lacked a clear structure for how to set up the work around the patients who used the app. The nurses themselves had to create a structure for how to monitor and respond to alerts, which took time from their usual tasks. Moreover, the experience was that that they spent more time on patients who used the app, which resulted in extra work. None of the units had adapted their workflow in any way when introducing the app, which meant that the resources available were the existing staff. The lack of structure, time, and staff resulted in work with the Interaktor system being deprioritized. However, there was a varying engagement in working with the system among the nurses, with different views on how to structure the work. Here is a discussion between 2 nurses:

The other day we talked about how we didn’t have any good routines for this system, or at least I felt that we didn’t have a routine for when to check for alerts, so that it just became automatic.

Hmm, but for myself, every time I walked by the phone, I checked it for alerts.

Two nurses at Unit C

The nurses mentioned that it was time-consuming to work with both the Interaktor system and the regular electronic medical records system. There was a strong wish that the two systems could have been integrated to a greater extent, so that information about the patients was easy to access when an alert came, and they could facilitate documentation of actions and advice.

#### Being Unsure About Multiple Ways to Interact With Patients

Often, when the nurses came to work on Mondays, there was a long list of alerts that had come in over the weekend. This created a concern that patients had not received help with their problems, and they felt obliged to call to make sure that no one was feeling unwell at home. Sometimes patients had awaited a call from the nurse, which made the nurses unsure about how to clarify ways to contact health care. Further, there was uncertainty as to whether alerts might have been missed or not, due to technical problems with the phone, or it not being checked regularly.

Since only a few patients were included in the study at any one time, the nurses felt that it was hard to set routines and keep track of patients who used the app. Most patients they cared for did not participate in the study and did not have access to the app, which made their work more difficult as different patients could reach them in different ways.

At a small unit, it was hard to respond to alerts within the specified times. Therefore, it happened that patients called the clinic’s advice line as well, which resulted in uncertainty as to whether the patient had already spoken to someone or not. In the same way, uncertainties arose about the responsibility to contact patients who were connected to the advanced home care team or who were about to receive chemotherapy at the treatment unit. As a result, the nurses found that patients received numerous phone calls and had contact with several nurses.

They meet a nurse in the treatment unit quite regularly and then maybe they feel that they do not need to report an alert. They know that tomorrow they can discuss this at the treatment unit. At least half of the alerts were connected to the treatment and then the nurses needed to talk to them to adjust the doses or something like that.

Nurse at Unit B

## Discussion

### Principal Results

Important findings in this study are that the nurses emphasized that incorporating mHealth in clinical practice is helpful in facilitating person-centered care. The Interaktor system enabled the nurses to form partnerships with patients and to capture patients in most need. However, they experienced obstacles in incorporating the Interaktor system due to lack of structural prerequisites and uncertainty about the multiple ways of interacting with patients.

### Limitations

There are some limitations with this study. For some periods of time, the nurses had few patients to monitor, which could well have affected their understanding of the system and its use. If other patient groups had been included in the study, the nurses might have gained more experience and insights about this new way to support and monitor patients with cancer. Further limitation is the risk that, when using focus groups, individual voices and certain views are prevented from emerging due to group dynamics [14]. Sometimes it was notable that someone expressed themselves more strongly than the others in the group. However, discrepancies were captured even at the same workplace, indicating that diverse views were given room in the interviews. A strength with the analysis is that the authors have different preunderstandings of the study and the data. The initial coding and analysis of themes was carried out without the influence of previous evaluations of the Interaktor app. Later in the analysis process, the researchers who conducted the interviews were involved, which facilitated the validation that initial thoughts were not lost during the analysis.

### Comparison With Prior Work

Forming partnerships with patients has been described as the foundation for person-centered care [16]. According to Swedish law and regulations, nurses are obligated to create conditions for individual planning, and to allow patients to participate in their own care and to perform self-care as much as possible [17,18]. The results illustrate several ways in which partnership and participation can be achieved by implementing the Interaktor app. The nurses had a close connection to patients, even if they were at home. Further, they could follow up on patients’ self-care through the symptom monitoring and then interact with patients and make individual plans. The results demonstrate that nurses are ready to use mHealth to bring knowledge and increase patients' participation in care, a focus that has been highlighted by international and national nurse associations [19,20].

One of the main findings, that the Interaktor system could capture patients in most need without patients having to be responsible for contacting health care themselves, is consistent with descriptions from patients using the app [12]. To be able to implement such a tool as a clinical routine, it is important that there is consensus on its benefits. On the other hand, when the responsibility was placed on the nurses, a worry arose that patients might be neglected. This highlights the importance of creating clear routines when implementing new ways of working.

Neither workplace had adapted their structure in any way prior to the introduction of the app, meaning that the nurses received this new workload in addition to their regular duties. Monitoring patients and responding to alerts took more time than the standard care, where patients got in touch with them when they needed something. The description of an extra workload has been highlighted previously in evaluations of mHealth and must be considered before implementation [21,22]. A clear structure, proper training, and introduction along with positive and engaged nurses are prerequisites when implementing mHealth [22]. Further, previous research has found that nurse managers have shown stronger motivation to use information and communication technology than registered nurses and that team climate and collegial and organizational support are essential to build positive experiences for health care professionals [23]. If the app is to be implemented in the standard care, it is important for workplace managers to adapt the workplace so that the app becomes part of the normal routine. This will require clear manuals and steering documents. Furthermore, focus needs to be placed on engaging nurses and making them identify benefits themselves, along with suitable training and introduction to the system.

Since the web interface was not incorporated in the systems that nurses already used daily, they experienced duplicated work, using two systems to document interactions and support to patients. This is in line with findings from a review where a major barrier for health care professionals to incorporate collection of PROs as clinical routine was that PROs were not incorporated in the hospital’s electronic records resulting in multiple log-ins with the risk of ineffectiveness [24]. For implementation work, it will be important to incorporate collection of PROs along with self-care advice and documentation of support into systems that are used daily.

The nurses pointed out that it was not optimal to call patients on Mondays for alerts that had occurred over the weekend. This was also brought up by patients as a flaw with the system, as it is often over weekends and during the night that feelings of loneliness can arise and thereby the need to talk to someone [12]. It has been concluded that a lack of outpatient services on weekends for patients following pancreatic cancer surgery leads to increased emergency room use, and that early identification and triage of adverse events are essential [25]. This highlights the need to offer patients with pancreatic cancer support outside of office hours, even for symptoms of less acute nature.

### Conclusions

In conclusion, the findings show that with the help of mHealth, health care professionals can gain more knowledge about patients at home and build relationships from a distance. As such, person-centered care can be facilitated for patients undergoing treatment for pancreatic cancer. By identifying patients in most need, health care professionals can allocate their resources accordingly, which makes care more effective. For tools such as Interaktor to be implemented in standard care, there are important organizational issues to consider. Health care professionals need time and resources to create new routines and adapt the workplace, together with proper introduction, training, and support from unit managers. Further, it would be beneficial if the app was incorporated in already-used systems and if alerts were monitored and responded to during all hours of the day.

## Abbreviations

mHealth: mobile health
PRO: patient-reported outcomes
